# Activity and Impact on Resistance Development of Two Antivirulence Fluoropyrimidine Drugs in *Pseudomonas aeruginosa*

**DOI:** 10.3389/fcimb.2019.00049

**Published:** 2019-03-11

**Authors:** Francesco Imperi, Ersilia V. Fiscarelli, Daniela Visaggio, Livia Leoni, Paolo Visca

**Affiliations:** ^1^Department of Science, Roma Tre University, Rome, Italy; ^2^Laboratory affiliated to Istituto Pasteur Italia–Fondazione Cenci Bolognetti, Department of Biology and Biotechnology Charles Darwin, Sapienza University of Rome, Rome, Italy; ^3^Laboratory of Cystic Fibrosis Microbiology, Bambino Gesú Hospital, Rome, Italy

**Keywords:** acquired resistance, antimetabolite, antivirulence drug, cystic fibrosis, *Pseudomonas aeruginosa*, siderophore, virulence

## Abstract

The rise in antibiotic resistance among bacterial pathogens has prompted the exploitation of alternative antibacterial strategies, such as antivirulence therapy. By inhibiting virulence traits, antivirulence drugs are expected to lessen pathogenicity without affecting bacterial growth, therefore avoiding the spread of resistance. However, some studies argued against this assumption, and the lack of antivirulence drugs in clinical use hampers the empirical assessment of this concept. Here we compared the mode of action and range of activity of two drugs which have been proposed for repurposing as quorum sensing and pyoverdine inhibitors in the human pathogen *Pseudomonas aeruginosa*: the anticancer drug 5-fluorouracil (5-FU) and the antimycotic drug 5-fluorocytosine (5-FC), respectively. The effect on bacterial growth, emergence and spread of resistance, and activity against clinical isolates were assessed. Our results confirm that 5-FU has growth inhibitory activity on reference strains and can rapidly select for spontaneous resistant mutants with loss-of-function mutations in the *upp* gene, responsible for uracil conversion into UMP. These mutants were also insensitive to the anti-pyoverdine effect of 5-FC. Conversely, 5-FC did not cause relevant growth inhibition, likely because of poor enzymatic conversion into 5-FU by cytosine deaminase. However, coculturing experiments showed that 5-FU resistant mutants can outcompete sensitive cells in mixed populations, in the presence of not only 5-FU but also 5-FC. Moreover, we observed that serial passages of wild-type cells in 5-FC-containing medium leads to the appearance and spread of 5-FC insensitive sub-populations of 5-FU resistant cells. The different effect on growth of 5-FU and 5-FC was overall conserved in a large collection of cystic fibrosis (CF) isolates, corresponding to different infection stages and antibiotic resistance profiles, although high variability was observed among strains. Notably, this analysis also revealed a significant number of pyoverdine-deficient isolates, whose proportion apparently increases over the course of the CF infection. This study demonstrates that the efficacy of an antivirulence drug with no apparent effect on growth can be significantly influenced by the emergence of insensitive mutants, and highlights the importance of the assessment of resistance-associated fitness cost and activity on clinical isolates for the development of “resistance-proof” antivirulence drugs.

## Introduction

Antibiotic resistance is a serious public health concern at the global level, as an alarmingly high level of drug resistance has been reported in most common bacterial pathogens (Tommasi et al., 2015), calling for the investigation of alternative therapeutic options. In the last decades, researchers started looking at virulence factors as targets for the development of novel anti-infective drugs aimed at inhibiting pathogen-dependent host damage rather than bacterial growth (Finlay and Falkow, 1997). Such molecules are referred to as antivirulence drugs (Rasko and Sperandio, 2010).

Traditional antibiotics hit essential cellular processes that are widely conserved among bacteria (Lange et al., 2007), imposing a strong selective pressure for resistant mutants and often causing damage and/or dysbiosis in the normal microbiota. Conversely, antivirulence drugs should target virulence-related traits which are typically pathogen-specific and not strictly required for bacterial growth. Hence, they are expected to exert negligible effects on commensal bacteria and to decrease the evolution rates toward resistance (Rasko and Sperandio, 2010). However, since antivirulence drugs still have to enter the cell and/or interact with specific molecular targets to exert their inhibitory activity, the existence of mechanisms conferring resistance to antivirulence compounds is predictable and, indeed, some of them have already been documented, such as modification of the target or extrusion of the antivirulence drug by efflux pumps (Shakhnovich et al., 2007; Maeda et al., 2012). Whether these mechanisms of resistance would be positively selected during the infection remains a matter of debate (García-Contreras et al., 2016; Russo et al., 2016). It has been proposed that the spread of mutant clones resistant to antivirulence drugs could depend on the “public availability” of the targeted virulence factor in the bacterial population. While shared (secreted) virulence factors are public goods that can be used by both sensitive and resistant subpopulations, individualized (cell-associated) virulence factors are private goods that benefit only the resistant producers, thus providing a selective advantage to the resistant subpopulation (reviewed in Allen et al., 2014; Ruer et al., 2015; Maura et al., 2016). Irrespective of the specific conditions, it is however commonly accepted that the selective pressure for resistance to antivirulence agents is likely much weaker than that for resistance to antibiotics (Maura et al., 2016). Unfortunately, the lack of antivirulence drugs in human or animal therapy does not allow the verification of these theories in a clinical or veterinary setting.

The present study was aimed at gaining further insights in the field of antivirulence drug discovery by comparing the mode of action, selective pressure toward resistance and range of activity of two fluorinated pyrimidine drugs, the anticancer drug 5-fluorouracil (5-FU) and the antimycotic drug 5-fluocytosine (5-FC), which have been proposed for repurposing as quorum sensing and pyoverdine inhibitors in the opportunistic human pathogen *Pseudomonas aeruginosa*, respectively. The antivirulence activity of 5-FU against *P. aeruginosa* was identified when it was found to counteract uracil-mediated activation of the quorum sensing response and to repress the expression of several virulence traits, including biofilm formation (Ueda et al., 2009). The antivirulence potential of fluorinated pyrimidines was later expanded by a drug repurposing screening campaign, which identified 5-FC as a potent inhibitor of pyoverdine siderophore production, and showed that this antimycotic drug can also suppress *P. aeruginosa* lethality in a mouse model of acute lung infection (Imperi et al., 2013), in line with the crucial role of pyoverdine-mediated iron uptake and virulence in this infection model (Minandri et al., 2016). The anti-*P. aeruginosa* efficacy of fluorinated pyrimidines was also supported by an *in vivo* screening in the *Caenorhabditis elegans* infection model, that revealed anti-pathogenic and anti-pyoverdine activities in 5-FU, 5-FC, and 5-fluorouridine (Kirienko et al., 2016). Notably, 5-FU had a broad inhibitory effect on several virulence phenotypes (Ueda et al., 2009), while 5-FC appeared to exert its antivirulence activity by targeting mainly the production of the pyoverdine siderophore and of pyoverdine-regulated virulence factors (Imperi et al., 2013; Kirienko et al., 2016). These works also reported that, while 5-FC does not affect *P. aeruginosa* growth even at high concentrations, 5-FU has a strong bacteriostatic effect on *P. aeruginosa*, in agreement with an older report describing the growth inhibitory activity of 5-FU, but not of 5-FC, against this bacterium (West, 1986).

Here we provide evidence that 5-FC and 5-FU likely share the same mechanism of action, and that the modest growth inhibitory activity of 5-FC is due to poor uptake and/or limited conversion into 5-FU by *P. aeruginosa* cytosine deaminase. By using co-culturing approaches and *in vitro* evolution experiments, we also demonstrated that 5-FC/5-FU insensitive spontaneous mutants with a defective pyrimidine salvage pathway readily emerge and spread in 5-FU treated populations and that, unexpectedly, these resistant mutants are also selected by 5-FC treatment, though at lower frequency. Finally, we found that the growth inhibitory and/or anti-pyoverdine activities of these two drugs are overall conserved in a large collection of cystic fibrosis (CF) isolates, although some inter-strain variability was observed.

## Materials and Methods

### Bacterial Strains, Growth Conditions, And Plasmids

Laboratory bacterial strains and plasmids used in this study are listed in Supplementary Table 1, while the 100 *P. aeruginosa* CF isolates analyzed in this work are described in Supplementary Table 2. The CF isolates belong to the collection of bacterial strains isolated from respiratory secretions (sputum, hypopharyngeal aspirate, bronchoalveolar lavage) of CF patients in follow-up at the Cystic Fibrosis Center of the Bambino Gesù Children's Hospital (Rome, Italy). Strain isolation and characterization were performed with the informed consent of the patients or of their parents/legal guardians for minors. Strains were grown in Lysogeny Broth, Lennox formulation (LB), or Mueller-Hinton (MH) as iron-rich media (Acumedia). The iron-depleted complex medium TSBD (Ohman et al., 1980) or the M9 minimal medium supplemented with 20 mM sodium succinate (SM9, Sambrook et al., 1989) were used as iron-poor media, to which FeCl_3_ was added at the indicated concentrations when required. Growth and pyoverdine assays were performed on bacteria cultured at 37°C in 96-well microtiter plates (200 μl of medium in each well) under static conditions, unless otherwise stated.

### Plasmid Construction

The plasmid pUCP*upp* was generated by cloning the coding sequence and the promoter region of the *upp* gene, previously amplified using the PAO1 genomic DNA as the template, into pUCP18. Primers and restriction sites used for PCR and gene cloning are listed in Supplementary Table 1.

The plasmid pUCP*codAcodB* was generated by extracting the coding sequence of *codA* from plasmid pUCP*codA* by BamHI-HindIII restriction. The fragment was blunt subcloned into pUCP18*codB* previously digested by SmaI. The correct fragment orientation was verified by restriction analysis. All constructs were verified by DNA sequencing.

### Growth and Pyoverdine Measurements

Growth was measured as the OD_600_ of bacterial cultures or of appropriate dilutions in sterile growth medium in a spectrophotometer or, when indicated, in a microtiter plate reader (Victor^2^V, Wallac). Unless otherwise stated, for growth and compound sensitivity assays bacteria were inoculated at ~10^6^ cells/ml from late exponential or early stationary phase cultures. In long-term resistance development assays, at each passage bacterial cultures were diluted 1:100 in fresh medium.

Pyoverdine production was measured as the OD_405_ of culture supernatants appropriately diluted in 0.1 M Tris-HCl (pH 8), using the supernatant of a pyoverdine-deficient mutant (PAO1Δ*pvdA*) as blank, and normalized to the OD_600_ of the corresponding culture (Imperi et al., 2010). Pyoverdine production by CF isolates was assessed fluorimetrically by recording the emission at 450 nm upon excitation at 400 nm (Imperi et al., 2009) of culture supernatants appropriately diluted in sterile growth medium, and normalized to the OD_600_ of the corresponding culture.

### Selection and Characterization of Spontaneous 5-FU Resistant Isolates

To obtain spontaneous 5-FU resistant isolates, *P. aeruginosa* PAO1 was cultured in LB until the late-exponential phase, normalized at ca. 2 × 10^9^ cells/ml in saline and 100-μl aliquots were plated onto SM9 agar plates containing 120 μg/ml 5-FU. Five microliters of serial 10-fold dilutions of the stock bacterial suspension were also spotted onto SM9 agar in the absence of 5-FU. Frequency of spontaneous 5-FU resistant mutants was calculated as the ratio between colony forming units (CFU)/ml obtained on 5-FU-containing plates and CFU/ml obtained on plates without 5-FU after 48 h of incubation at 37°C.

To check the DNA sequence of the *upp* gene in selected 5-FU-resistant mutants, their genomic DNA was extracted using GenElute Bacterial Genomic DNA kit (Sigma-Aldrich) and used as the template for a PCR with primers external to the *upp* gene (Supplementary Table 1), which were also used for sequencing of both DNA strands.

### Statistical Analyses

Statistical analysis was performed with the software GraphPad Instat, using either using One-Way Analysis of Variance (ANOVA) followed by Tukey-Kramer multiple comparison tests or the Kruskal–Wallis test followed by uncorrected Dunn's multiple comparison test.

## Results

### Both 5-FC and 5-FU Reduce Pyoverdine Production but Only 5-FU Causes Relevant Growth Inhibition

**Figure 1:**
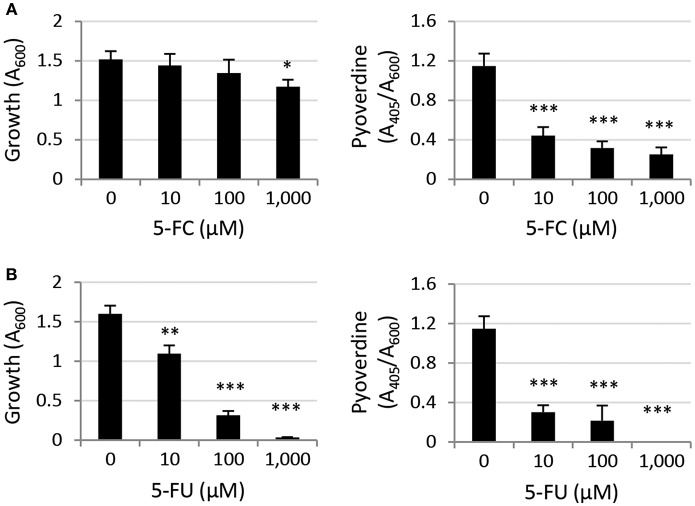
Effect of **(A)** 5-fluorocytosine (5-FC) and **(B)** 5-fluorouracil (5-FU) (0–1,000 μM) on *P. aeruginosa* PAO1 growth (left panels) and relative pyoverdine production (right panels) after 14-h growth at 37°C in the iron-poor complex medium TSBD. Values represent the mean (±SD) of six independent assays. Asterisks indicate statistically significant differences with respect to the untreated control (*P < 0.05, **P < 0.01, ***P < 0.001; ANOVA).

As a first attempt to compare the activity of the two antimetabolite drugs 5-FC and 5-FU we assessed their effect on growth and pyoverdine production of the reference strain *P. aeruginosa* PAO1 in the iron-poor complex medium TSBD which was originally used by our group in the screening of anti-pyoverdine activity among FDA-approved drugs (Imperi et al., 2013). In line with the results recently obtained by the Kirienko's group using NGM (nematode growth medium) (Kirienko et al., 2016), we confirmed that 5-FC has poor growth inhibitory activity at concentrations up to 1 mM, while 5-FU strongly inhibits *P. aeruginosa* PAO1 growth between 100 and 1,000 μM (Figure 1). Both compounds caused strong reduction of pyoverdine production at all concentrations tested (≥10 μM; Figure 1).

To confirm that the differential effect of 5-FC and 5-FU on *P. aeruginosa* PAO1 growth was not specific to the iron-poor culture medium used to monitor pyoverdine production, we also performed growth inhibition assays in different media, including complex iron-rich media (MH and LB), the minimal medium SM9 and, as control, TSBD. Growth was measured in a microtiter plate reader after 20 h at 37°C under static conditions, in order to determine IC_90_ values (compound concentrations needed to cause 90% growth inhibition). As shown in Supplementary Figure 1, 5-FC did not cause relevant alteration of *P. aeruginosa* PAO1 growth in any tested medium. In contrast, 5-FU inhibited growth in all media, although the inhibitory effect varied depending on the medium, with IC_90_ values ranging from 20 μM in the minimal medium SM9 to 625–2,500 μM in complex media (Supplementary Figure 1).

### Poor Conversion of 5-FC Into 5-FU Is Responsible for the Lack of Growth-Inhibitory Activity Of 5-FC

**Figure 2:**
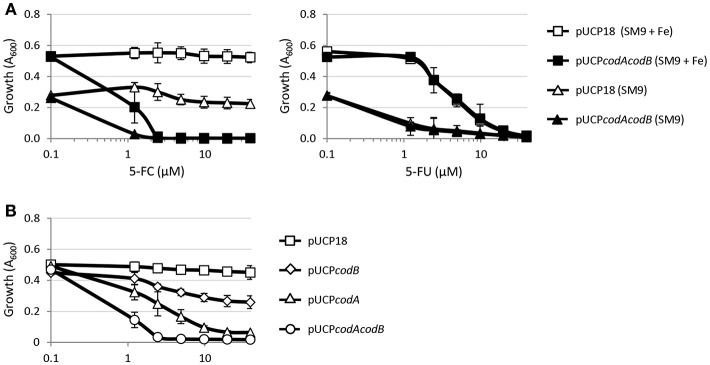
**(A)** Effect of 5-fluorocytosine (5-FC, left panel) or 5-fluorouracil (5-FU, right panel) at the indicated concentrations on the growth of *P. aeruginosa* PAO1 pUCP18 or *P. aeruginosa* PAO1 pUCP*codAcodB* in SM9 minimal medium, supplemented or not with 100 μM FeCl_3_ (+ Fe), after 18-h incubation in microtiter plates at 37°C. **(B)** Effect of 5-FC on the growth of *P. aeruginosa* PAO1 harboring pUCP18, pUCP*codA*, pUCP*codB*, or pUCP*codAcodB* in SM9 with 100 μM FeCl_3_ after 18-h incubation in microtiter plates at 37°C. Growth (OD_600_) was measured in a microtiter plate reader, and values represent the mean (±SD) of three independent assays.

We have previously demonstrated that the anti-pyoverdine activity of 5-FC relies on its metabolic conversion into 5-FU in the cytoplasm of *P. aeruginosa* cells, which requires the concomitant activity of cytosine permease CodB and cytosine deaminase CodA, involved in 5-FC transport and conversion into 5-FU, respectively (Imperi et al., 2013). The finding that, differently from 5-FU, 5-FC has marginal (if any) activity on growth (Figure 1; Kirienko et al., 2016) suggests that the uptake of 5-FC and/or its conversion into 5-FU could represent the limiting step(s) for its antibacterial activity. To explore this hypothesis, we investigated the effect of CodAB overexpression on 5-FC sensitivity in the wild type PAO1. As reported in Figure 2A, CodAB-overexpressing cells showed a marked sensitivity to 5-FC, which completely inhibited bacterial growth in SM9 minimal medium at low μM concentrations. As expected, CodAB overexpression did not influence the activity of 5-FU (Figure 2A), indicating that the observed increase in the growth inhibitory effect of 5-FC was not related to a general increase in sensitivity to fluorinated pyrimidine analogs. Notably, individual overexpression of CodA and, to a lesser extent, of CodB also increased *P. aeruginosa* sensitivity to 5-FC, even if the effect was much lower than that caused by CodAB overexpression (Figure 2B). These results indirectly imply that exogenously provided 5-FC is poorly converted into 5-FU by *P. aeruginosa* PAO1 cells, hence suggesting that the differential antivirulence and growth inhibitory effect of 5-FU is likely dependent on the intracellular concentration of this antimetabolite.

### Spontaneous 5-FU Resistant Mutants Acquire Mutations in the *upp* Gene and Are Also Insensitive to the Anti-pyoverdine Activity Of 5-FC

**Figure 3:**
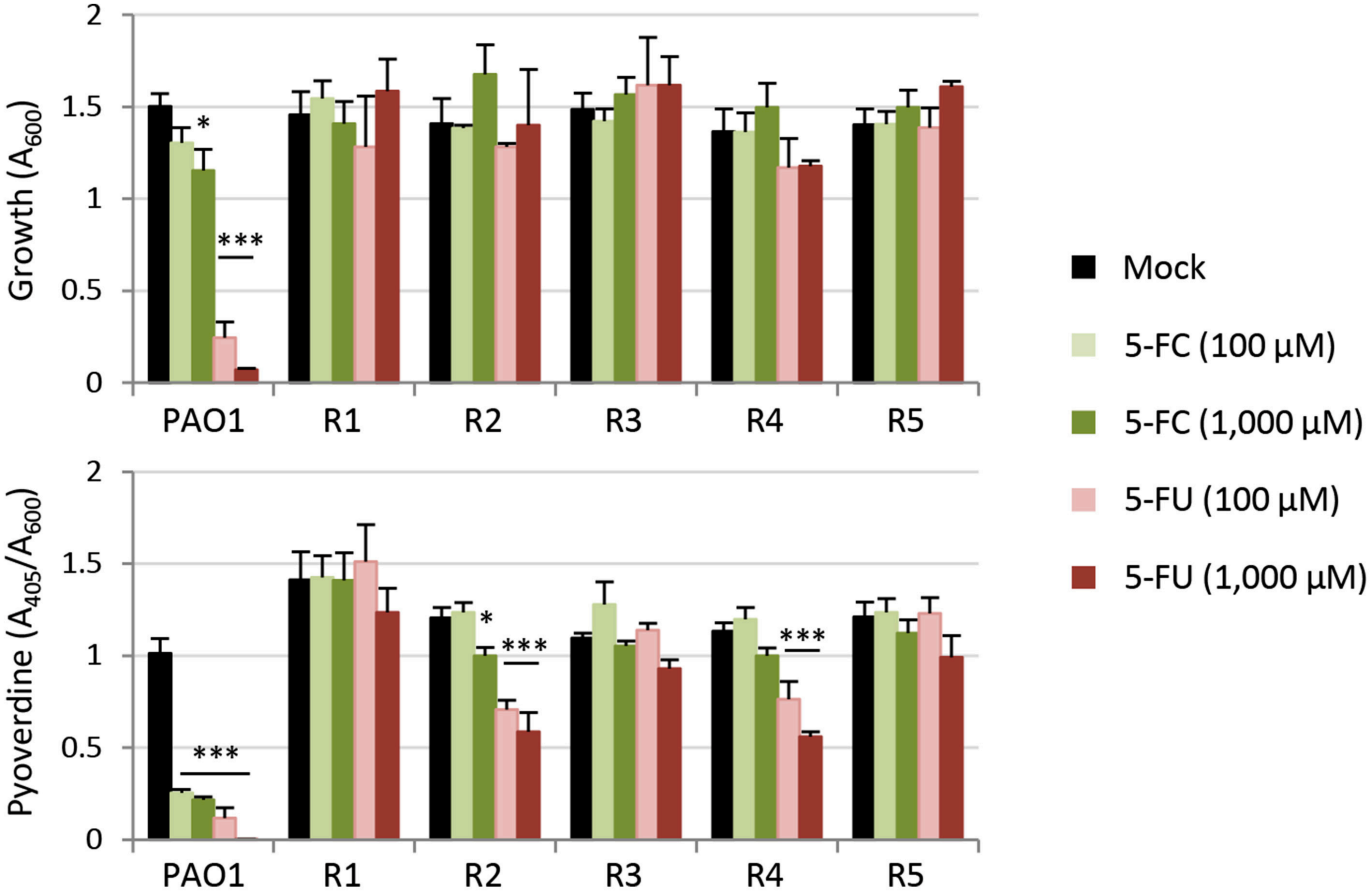
Effect of 5-fluorocytosine (5-FC) and 5-fluorouracil (5-FU) (0–1,000 μM) on growth and relative pyoverdine production (upper and lower panel, respectively) of *P. aeruginosa* PAO1 and five isogenic spontaneous mutants resistant to 5-FU (R1–R5) cultured in TSBD for 14 h in microtiter plates at 37°C. Values represent the mean (±SD) of three independent experiments. Asterisks indicate statistically significant differences with respect to the corresponding untreated control (*P < 0.05, ***P < 0.001; ANOVA).

Taking advantage of the strong inhibitory effect of 5-FU on *P. aeruginosa* PAO1 growth in the minimal medium SM9 (Supplementary Figure 1), we used SM9 agar plates supplemented with 120 μM 5-FU (6 × IC_90_) to select for spontaneous mutants resistant to this drug. In five independent experiments, we obtained 5-FU resistant isolates with an average frequency of 1.3 (±0.7) × 10^−7^. When streaked on SM9 agar plates containing either 100 μM 5-FC or 100 μM 5-FU, all colonies appeared to be not only resistant to 5-FU but also insensitive to the inhibitory activity of 5-FC on pyoverdine production, which was visually assessed as fluorescence under the UV light (data not shown; Visaggio et al., 2015). To confirm this qualitative observation, we selected five spontaneous mutants (obtained in independent assays) and quantitatively assessed their growth and pyoverdine production profile in the presence of high concentrations of 5-FU or 5-FC (100 and 1,000 μM) (Figure 3). All mutants were found to be resistant to the growth inhibitory effect of 5-FU as well as to the anti-pyoverdine activity of both drugs, although pyoverdine production was still partly repressed by 5-FU in the spontaneous mutants R2 and R4 (Figure 3).

**Figure 4:**
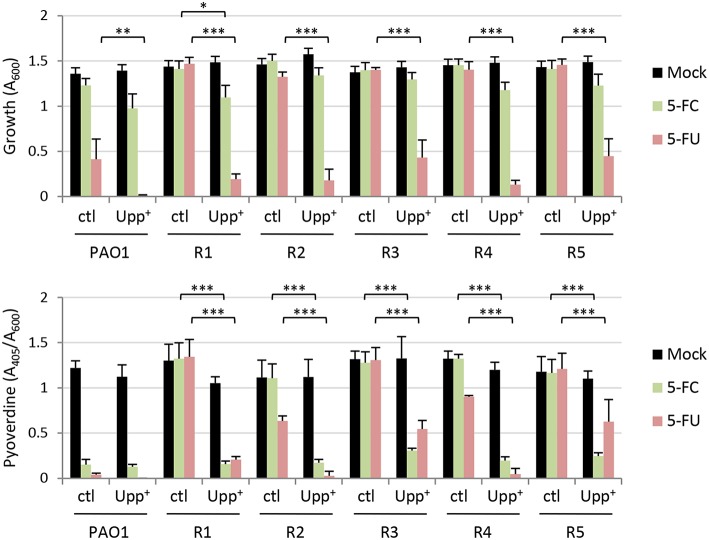
Effect of 5-fluorocytosine (5-FC) and 5-fluorouracil (5-FU) supplemented at 100 μM on growth and relative pyoverdine production (upper and lower panel, respectively) of *P. aeruginosa* PAO1 and five isogenic spontaneous mutants resistant to 5-FU (R1–R5), containing the empty plasmid pUCP18 (ctl) or the Upp-expressing construct pUCP*upp* (Upp^+^). Measurements were taken after 14-h growth in TSBD in microtiter plates at 37°C. Values represent the mean (±SD) of three independent experiments. Asterisks indicate statistically significant differences with respect to the corresponding control carrying the empty plasmid and cultured under the same condition (*P < 0.05, **P < 0.01, ***P < 0.001; ANOVA).

Very recently it has been shown that *P. aeruginosa* mutants resistant to 5-FC spontaneously arise after long-term treatment with 5-FC in human serum, and whole genome sequencing revealed that these mutants invariably carried mutations in the *upp* gene (Rezzoagli et al., 2018), which encodes the uracil phosphoribosyltransferase responsible for conversion of uracil into the nucleotide precursor UMP in the pyrimidine salvage pathway (Beck and O'Donovan, 2008). To verify whether *upp* mutations were also present in our spontaneous 5-FU resistant mutants, we sequenced the entire *upp* gene, including its promoter. A nonsense point mutation was identified in the isolate R5, causing premature translation termination at codon 57 (of 213). Missense point mutations were identified in isolates R1, R2, and R4, leading to the amino acid substitution H173P (R1) or H8P (R2 and R4, carrying an identical mutation although they were selected in independent assays) (Supplementary Table 1). For the remaining clone (R3), several attempts to amplify the *upp* gene with different primer pairs failed, and amplification and sequencing of the *upp* genomic locus revealed a ca. 5-kb deletion event involving the *upp* gene and the neighboring genes PA4642-PA4645, *uraA, cupE1*, and part of *cupE2* (Supplementary Figure 2). Notably, codons 8 and 173 correspond to hotspot mutation sites that were identified in the *Salmonella enterica upp* gene of *in vitro*-selected 5-FU resistant mutants (Glaab et al., 2005), and encode for histidine residues that are highly conserved in Upp proteins from eubacterial species (Supplementary Figure 3), arguing for an important role of such residues in the functionality of the enzyme. To verify this hypothesis and, thus, to tentatively correlate 5-FU resistance with the loss of function of Upp, we complemented the 5-FU resistant mutants with a plasmid expressing the wild type copy of the *upp* gene, and determined the effect on 5-FU and 5-FC sensitivity. Overall, the expression of a functional Upp protein in 5-FU resistant mutants almost completely restored their sensitivity to 5-FU and also rescued the anti-pyoverdine activity of 5-FC, indirectly confirming that the mutations identified in the *upp* gene of these isolates likely result in the loss of Upp function (Figure 4).

### 5-FU Resistant Mutants Are Positively Selected in the Presence of Clinically-Relevant Concentrations of Both 5-FU And 5-FC

**Figure 5:**
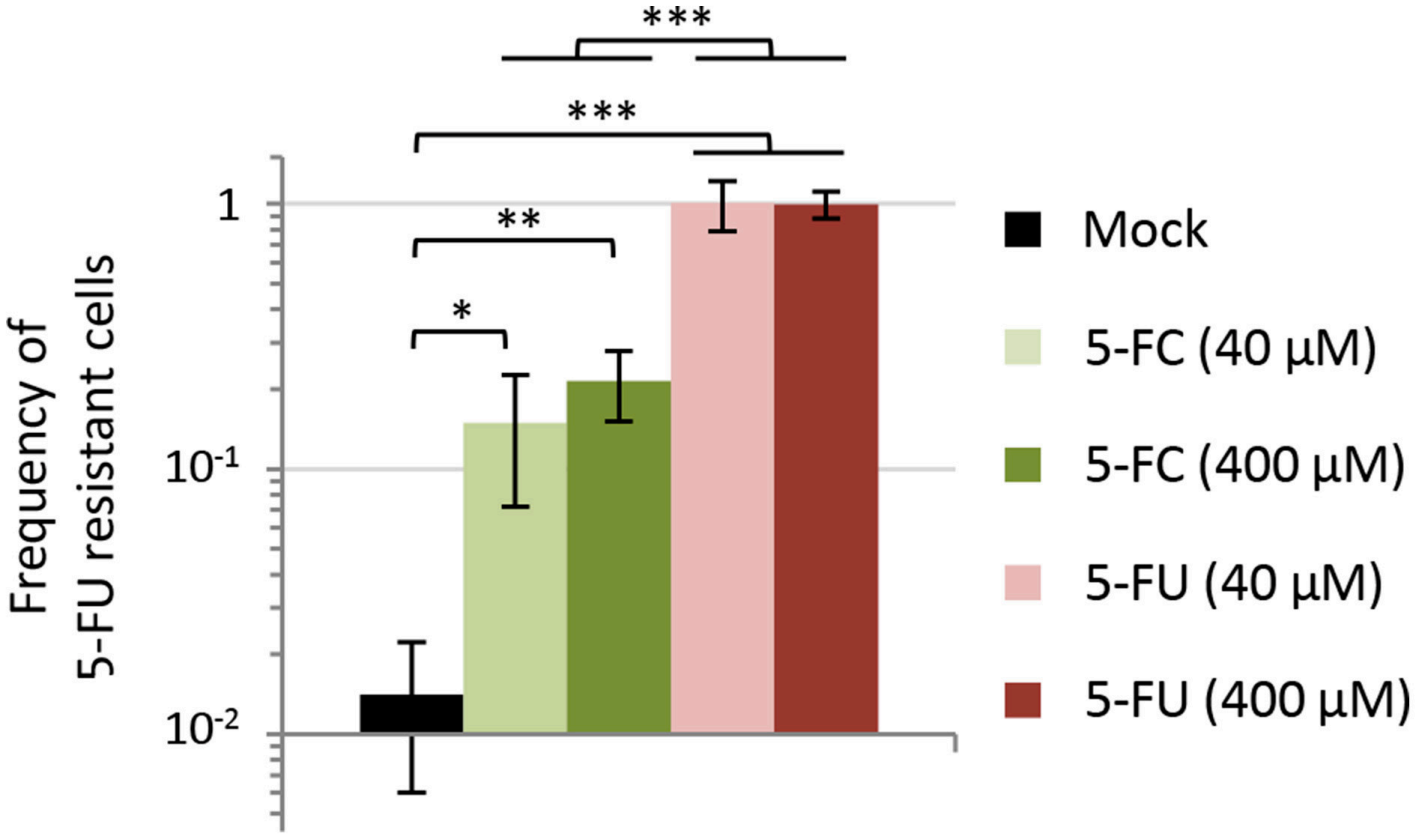
Effect of 5-fluorocytosine (5-FC) and 5-fluorouracil (5-FU) at 40 or 400 μM on the competitive fitness of spontaneous 5-FU resistant mutants of *P. aeruginosa* PAO1. Each 5-FU resistant isolate (R1, R2, R3, R4, or R5) and the parental strain PAO1 were precultured in TSBD with 50 μM FeCl_3_ and then inoculated at 1:1,000 final dilution in TSBD in a 1:100 ratio (5-FU R:WT). Percentage of 5-FU resistant cells with respect to 5-FU sensitive ones was evaluated after 16-h incubation at 37°C by plating on SM9 agar plates with or without 120 μM 5-FU. Values represent the mean (±SD) of ten independent experiments (two for each 5-FU resistant isolate). Asterisks indicate statistically significant differences (*P < 0.05, **P < 0.01, ***P < 0.001; ANOVA).

The availability of spontaneous 5-FU resistant isolates that were also insensitive to the anti-pyoverdine effect of 5-FC gave us the opportunity to assess whether the genetic events leading to 5-FC insensitivity could be selected for during 5-FC treatment. As a first attempt to investigate this issue, we co-cultured the wild type PAO1 and each spontaneous resistant isolate (R1–R5) in a 100:1 ratio in the iron-poor medium TSBD in the presence or absence of 5-FC at 400 μM, corresponding to the maximum serum concentration of 5-FC that is well-tolerated in humans (50 μg/mL, ca. 390 μM; Vermes et al., 2000), and a 10-fold lower concentration (40 μM). When the cultures reached the stationary phase (after ~10 generations), the percentage of 5-FU resistant cells with respect to 5-FU sensitive cells was determined through serial dilution plating on SM9 agar plates containing or not 120 μM 5-FU. Notably, 5-FU resistant cells rose from 1% to about 10 and 15% in the presence of 40 and 400 μM 5-FC, respectively (Figure 5). As control, the same experiment was also performed with equivalent concentrations of 5-FU. In line with the strong growth inhibitory activity of 5-FU (Figures 1, 2; Kirienko et al., 2016), 5-FU resistant cells completely outcompeted wild type ones in the presence of both 5-FU concentrations (Figure 5). The percentage of 5-FU resistant cells remained nearly constant (ca. 1.4%) in the absence of drugs (Figure 5), indicating that the increased fitness of 5-FU resistant cells observed in the presence of 5-FC was actually due to their competitive advantage over wild type (sensitive) cells.

**Figure 6:**
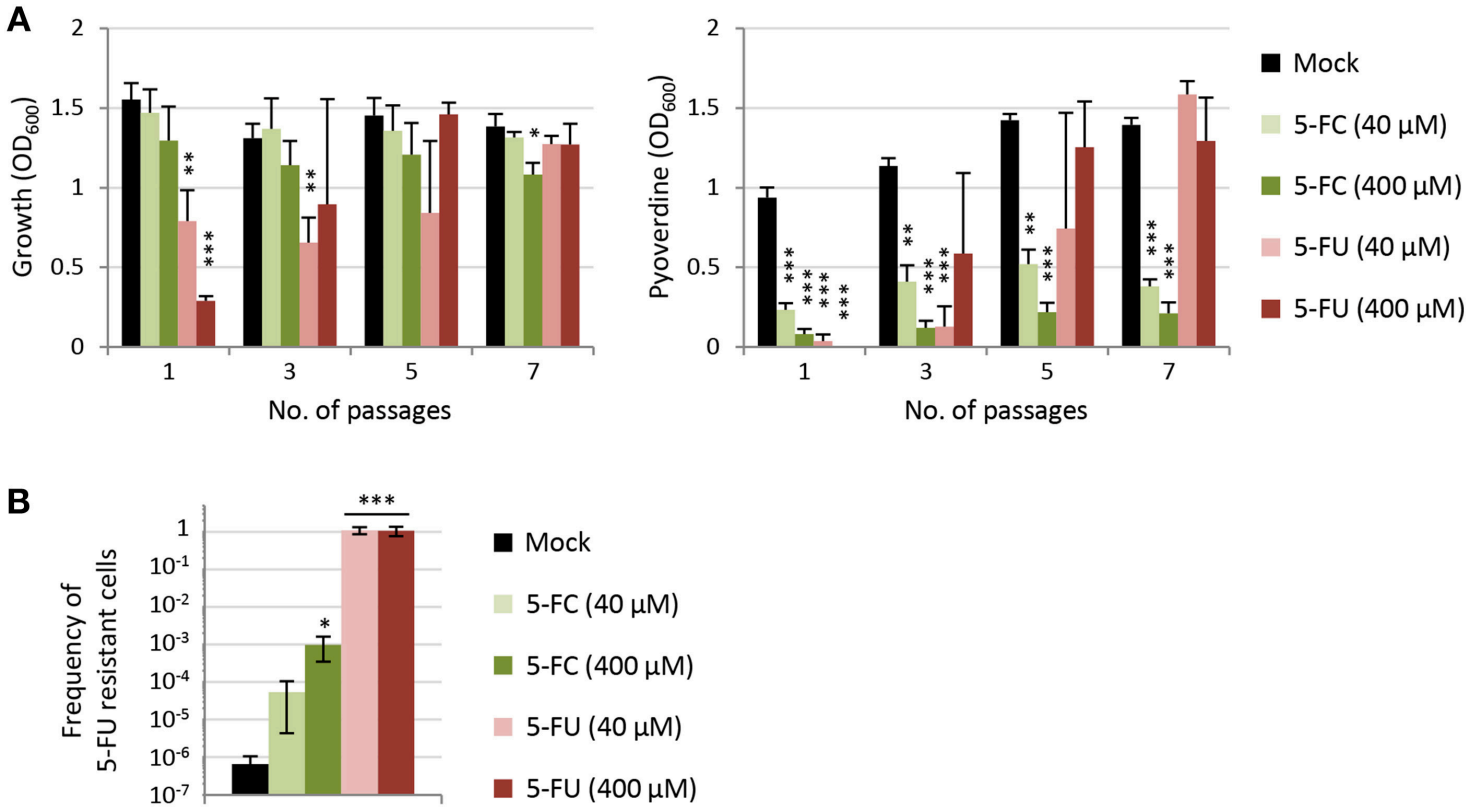
**(A)** Effect of 5-fluorocytosine (5-FC) and 5-fluorouracil (5-FU) on growth and relative pyoverdine production (left and right panel, respectively) of *P. aeruginosa* PAO1 cultured for 7 subsequent passages (1:100 dilution each) in TSBD in the presence or absence of 5-FC or 5-FU at 40 or 400 μM. **(B)** Frequency of 5-FU resistant cells after 7 passages in TSBD in the presence or in the absence of 5-FC or 5-FU at 40 or 400 μM. Values represent the mean (±SD) of three independent experiments. Asterisks indicate statistically significant differences with respect to the corresponding untreated control (*P < 0.05, **P < 0.01, ***P < 0.001; ANOVA).

The above experiment indicates that, when a relatively abundant number of 5-FC insensitive cells (1%) are artificially introduced in the population, they are positively selected by 5-FC treatments. To corroborate this finding in a more natural setting, a long-term experiment was performed by sequentially culturing the wild type PAO1 in TSBD containing 40 or 400 μM 5-FC or 5-FU for 7 passages (each involving almost 7 generations, for a total of ca. 46 generations). Growth and pyoverdine production were monitored over passages, while the percentage of 5-FU resistant cells was determined at the end of the experiment (Figure 6). This assay was aimed at verifying whether the 5-FU insensitive cells emerging in the population are positively selected by the drug treatment. As expected, over the passages the cultures treated with 5-FU became progressively more resistant to this drug. After 7 passages, the cultures exposed to 5-FU showed growth and pyoverdine levels comparable to untreated cultures, irrespective of the 5-FU concentration used in subcultures (Figure 6A), and accordingly they contained only 5-FU resistant cells (Figure 6B). Conversely, sensitivity to the anti-pyoverdine effect of 5-FC remained overall constant over the course of the experiment for cultures treated with this drug, irrespective of the concentration used (Figure 6A). However, long-term exposure to 5-FC led to an 80-fold or a 1,500-fold increase in the frequency of 5-FU resistant cells in the bacterial populations as compared to untreated cultures (Figure 6B). At the end of our assay, 5-FU resistant cells still represented ≤0.1% of the whole population, and for this reason they did not (yet) affect the efficacy of 5-FC in inhibiting pyoverdine production by the bacterial culture. Nevertheless, overall these results clearly indicate that, at clinically meaningful concentrations, 5-FC can exert a selective pressure in the TSBD medium which is much lower than that exerted by 5-FU, but still enough to promote the emergence of 5-FC insensitive subpopulations.

### The Differential Activity of 5-FC and 5-FU Is Overall Conserved in a Collection of *P. aeruginosa* CF Isolates

An important step in the development of any antibacterial drug is the evaluation of its activity on large panels of clinical strains. Since fluoropyridines have potential application as antivirulence adjuvants in treatment of *P. aeruginosa* infection in CF patients (Costabile et al., 2016), we compared the growth inhibitory and anti-pyoverdine effects of 5-FC and 5-FU at two different concentrations (10 and 100 μM) on 100 *P. aeruginosa* clinical strains isolated from the lung of CF patients. The collection included sequential isolates obtained during several years after establishment of the lung infection and showing different antibiotic resistance profiles (Supplementary Table 2), in order to verify whether the long-lasting *in vivo* adaption of *P. aeruginosa* to the CF lung and/or the acquisition of resistance to conventional antibiotics could affect the efficacy of the antivirulence drugs 5-FC and 5-FU.

Three isolates did not grow in the iron-depleted medium used to assess pyoverdine production (TSBD) (Supplementary Table 2) and, for this reason, were not included in the analysis. Moreover, a relevant percentage of CF isolates (17.5%) did not produce detectable amounts of pyoverdine under the conditions tested, and were therefore considered as pyoverdine-deficient strains in this work (Supplementary Table 2). Notably, the percentage of these pyoverdine-deficient isolates appeared to increase over years of CF lung colonization (Supplementary Figure 4).

**Figure 7:**
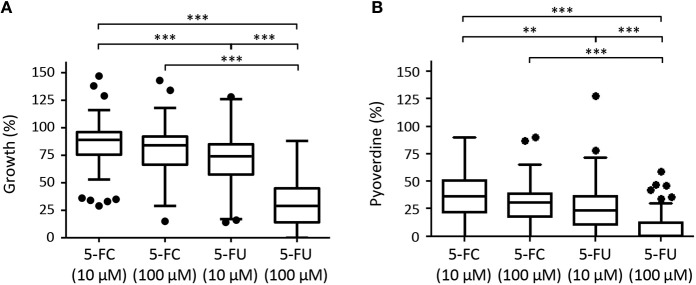
Box plots with Turkey whiskers showing the effect of 5-fluorocytosine (5-FC) and 5-fluorouracil (5-FU) at 10 or 100 μM on growth **(A)** and pyoverdine production **(B)** in a collection of 97 **(A)** or 80 **(B)** *P. aeruginosa* CF isolates cultured in TSBD until the late-exponential growth phase. Data are expressed as percentage with respect to untreated cultures (100%). For each isolates, values are the mean of two or three independent assays. Of the 100 isolates originally included in the collection, 3 did not grow and 17 did not produce detectable amounts of pyoverdine under the conditions tested, and were not included in the plots shown in **(A,B)**, respectively. Black dots represent the outliers. Asterisks indicate statistically significant differences (**P < 0.01, ***P < 0.001; Kruskal-Wallis).

Although high variability in both antivirulence and growth inhibitory activities was observed among strains, CF isolates were found to be overall sensitive to the anti-pyoverdine activity of both 5-FC and 5-FU (Figure 7). However, while 5-FC had moderate effect on the growth (<30% reduction) of most (≥70%) isolates, and no significant differences were observed between 10 and 100 μM 5-FC treatments, 5-FU caused a strong inhibition of growth in a dose-dependent manner, with >50% growth reduction for the majority (80%) of isolates at 100 μM concentration (Figure 7, Supplementary Table 2), in accordance with the results obtained for the reference strain PAO1 (Figure 1). For the great majority of isolates, a good correlation was observed between susceptibility to the growth inhibitory effect of 5-FU and susceptibility to the anti-pyoverdine activity of 5-FC (Supplementary Table 2), further confirming that the modes of action of these drugs are intimately linked in *P. aeruginosa*. However, some exceptions were noted, such as isolates that were highly sensitive to the anti-pyoverdine activity of 5-FC but less susceptible to growth inhibition by 5-FU (e.g., isolates BG17, BG79, and BG84), and isolates showing high growth inhibition but low pyoverdine reduction in the presence of 5-FU and/or 5-FC (e.g., isolates BG49 and BG55) (Supplementary Table 2).

When CF isolates were grouped according to the duration of CF lung colonization or of their antibiotic resistance profile no statistically-significant differences were observed among groups for 5-FC treatments (Supplementary Figure 5), suggesting that the adaptation of *P. aeruginosa* to the CF lung and the expression of antibiotic resistance determinants have no impact on 5-FC sensitivity. Similar results were overall obtained for 5-FU, although isolates from early stages of chronic colonization showed slightly higher sensitivity to 5-FU (Supplementary Figure 5).

## Discussion

By definition, antivirulence drugs are compounds that should suppress virulence phenotypes without affecting bacterial growth. This paradigm has been used in the last 15 years to search for antivirulence activities in natural or synthetic compounds, as well as in drugs already used for other clinical purposes, for their repositioning as antivirulence agents (Rangel-Vega et al., 2015; Mühlen and Dersch, 2016; Silva et al., 2016; Dickey et al., 2017; Johnson and Abramovitch, 2017; Rampioni et al., 2017a). Hundreds of antivirulence compounds have been proposed so far, and for most of them it has been demonstrated that they actually inhibit pathogenic traits at concentrations that have no impact on bacterial growth and cell viability, at least *in vitro*. The selectivity toward virulence is indispensable to ensure that antivirulence drugs provide an important advantage over traditional antibiotics, i.e., the lower selective pressure toward the emergence and spread of drug resistance. However, relatively few studies have actually explored the possible acquisition of resistance to antivirulence drugs and/or the effect of such resistance on the efficacy of antivirulence treatments.

To investigate this aspect, in this work we analyzed two highly-related antivirulence drugs endowed with significant antivirulence activity and different impact on *P. aeruginosa* growth, namely the fluorinated pyrimidine analogs 5-FU and 5-FC (Figure 1, Supplementary Figure 1; Kirienko et al., 2016). The growth inhibitory activity of 5-FU allowed us to easily select spontaneous 5-FU-resistant mutants, which were characterized by loss-of-function mutations in the *upp* gene, responsible for conversion of uracil into the nucleotide precursor UMP in the pyrimidine salvage pathway (Figures 3, 4, Supplementary Table 1; Beck and O'Donovan, 2008). This implies that the growth inhibitory activity of 5-FU is mainly related to inhibition of nucleic acid synthesis, as it has been proposed to occur in yeasts and cancer cells (Vermes et al., 2000; Longley et al., 2003). Interestingly, 5-FU-resistant mutants also became resistant to the anti-pyoverdine activity of 5-FC (Figure 3). This finding is not new, as mutations in the *upp* gene were recently identified through whole genome sequencing in 5-FC insensitive clones of *P. aeruginosa* selected during 5-FC treatment after subsequent passages in human serum (Rezzoagli et al., 2018), a medium in which iron uptake mediated by pyoverdine is important for growth (Bonchi et al., 2015). The evidence that Upp dysfunction confers resistance to both 5-FU and 5-FC strongly suggests that these two drugs likely share the same mechanism(s) of action. Accordingly, we found that 5-FC becomes highly toxic to *P. aeruginosa* cells which ectopically over-express both CodB and CodA or CodA alone (Figure 2), implying that poor cytoplasmic conversion of 5-FC into 5-FU is responsible for the modest growth inhibitory effect of 5-FC.

To date, the molecular target(s) and mechanism(s) of action allowing 5-FU metabolic derivatives to exert their antibacterial or anti-pyoverdine activity are still unknown. It could be hypothesized that fluorinated ribonucleotides have a different impact on the transcription and/or translation of different subsets of genes, with some virulence genes (e.g., pyoverdine genes) being more sensitive to inhibition of gene expression than housekeeping genes, thus explaining the antivirulence activity of these compounds at intracellular concentrations that do not affect growth. Such kind of effect has been proposed to explain the selective inhibition of some virulence genes by azithromycin at sub-MIC concentrations in *P. aeruginosa* (reviewed in Imperi et al., 2014). An alternative hypothesis is that fluorinated ribonucleotides could cause a general imbalance in the metabolic fluxes of the cell, which would be proportional to the intracellular concentration of the toxic compounds. At sub-inhibitory concentrations, this could result in the redistribution of cell energy and metabolites toward essential functions at the expense of non-essential processes, such as the energy-demanding biosynthesis of the pyoverdine siderophore (Visca et al., 2007), in order to ensure cell viability and reproduction. Although some reports have indeed highlighted some direct or indirect effects of catabolism and/or nutrient availability on virulence gene expression in *P. aeruginosa* (Linares et al., 2010; Yeung and Hancock, 2011; Raneri et al., 2018), further studies are still needed to verify and characterize the possible link between the metabolic state of the cell and its virulence potential.

What was unexpected is the finding that, when 5-FU/5-FC resistant mutants were artificially mixed with a population of sensitive cells in a culture medium (TSBD) in which 5-FC has basically no growth inhibitory effect and pyoverdine is dispensable for growth (Figure 1, Supplementary Figure 1; Imperi et al., 2013), these mutants were positively selected in the presence of 5-FC (Figure 5). Such evidence was corroborated by subsequent passages of the wild type strain PAO1 in the presence of 5-FC, which led to the emergence and spread of small sub-populations of 5-FC insensitive cells (Figure 6). This result clearly indicates that 5-FC may exert some selective pressure for resistance, even under conditions where it has no apparent effect on growth. Whether this might be due to subtle effects of 5-FC on metabolism or to a competitive advantage of cells with an impaired pyrimidine salvage pathway (*upp* mutants) over parental cells under the specific conditions tested remains to be determined. It must be remarked, however, that 5-FC/5-FU resistant mutants are much more readily selected by 5-FU treatments than by 5-FC treatments (Figures 5, 6), in line with the relevant growth inhibition caused by 5-FU (Figure 1 and Supplementary Figure 1). This provides a direct evidence that the emergence and spread of drug insensitive populations are proportional to the growth inhibitory activity of the drug (Maura et al., 2016).

With respect to the highly-conserved and constitutively-expressed antibiotic targets, the presence, expression levels and relevance to infection of virulence factors are often strain-dependent. This is especially true for opportunistic pathogens, such as *P. aeruginosa*, in which the virulence potential is multifactorial and combinatorial, implying that different combinations of pathogenic traits are important for virulence in different strains and/or in different infection models (Lee et al., 2006; Dubern et al., 2015). Thus, an important aspect of antivirulence drug development should be the evaluation of the range of antivirulence activity on large panels of clinical isolates (García-Contreras et al., 2013; Rampioni et al., 2017a,b). As demonstrated in this work using a large collection of CF isolates, the growth and pyoverdine inhibitory effects of 5-FC and 5-FU is overall conserved for clinical isolates (Figure 7), even if few strains revealed peculiar behaviors, such as low responsiveness to one drug and high sensitivity to the other (Supplementary Table 2). Even though these exceptions were not further investigated in this work, they suggest that the activity of 5-FC and 5-FU could be at least partly decoupled in some genetic backgrounds. Finally, it should be noted that a relatively high percentage of the CF isolates (17.5%) were found to be defective in pyoverdine production. As expected, pyoverdine deficiency was more prevalent among isolates obtained at late stages of chronic infection (Supplementary Figure 4), in accordance with previous reports showing that *P. aeruginosa* evolves in the CF lung toward less virulent phenotypes and/or the use of iron sources alternative to pyoverdine (De Vos et al., 2001; Marvig et al., 2014, 2015). However, pyoverdine-deficient strains also represented almost 10% of CF isolates from early stages of infection (Supplementary Figure 4). Given that these isolates are likely to be insensitive to the antivirulence activity of 5-FC, this evidence suggests that the treatment with 5-FC could have no (or poor) efficacy in a relevant number of CF patients.

In conclusion, this work clearly demonstrates that the lack of evident growth inhibitory activity in a putative antivirulence drug is not sufficient to rule out that resistance mechanisms can emerge and spread in the bacterial population, even under *in vitro* conditions in which the targeted virulence factor(s) is not required for growth. Although it might be argued that our results could represent a specific case related to the drugs (i.e., fluorinated pyrimidines) used in the present work, it is important to note that the number of studies proposing antimetabolites or even antibiotics at sub-MIC concentrations as potential antivirulence compounds is constantly increasing (Rangel-Vega et al., 2015; Mühlen and Dersch, 2016; Silva et al., 2016; Johnson and Abramovitch, 2017; Rampioni et al., 2017a; Soo et al., 2017; D'Angelo et al., 2018). In our opinion, the isolation of drug-insensitive spontaneous mutants, either by direct selection in the presence of high (inhibitory) drug concentrations or by means of more sophisticated culturing approaches to mimic conditions in which the targeted virulence factor(s) is important for growth (Maeda et al., 2012; Rezzoagli et al., 2018), and the assessment of the fitness of these resistant mutants during drug exposure should be mandatory tests in the antivirulence research field. This would ultimately increase the chance to identify and develop antivirulence drugs that are nearly “resistance-proof.”

## Data Availability

All datasets generated for this study are included in the manuscript and/or the supplementary files.

## Author Contributions

FI and PV conceived and designed experiments and wrote the paper. FI and DV performed the experiments. FI, EF, LL, and PV analyzed the data and contributed reagents, materials, and analysis tools. All authors read and approved the final manuscript.

### Conflict of Interest Statement

The authors declare that the research was conducted in the absence of any commercial or financial relationships that could be construed as a potential conflict of interest.
