# Supplementary material for: Activity and Impact on Resistance Development of Two Antivirulence Fluoropyrimidine Drugs in Pseudomonas aeruginosa
Source: Front Cell Infect Microbiol. 2019 Mar 11;9:49. doi: 10.3389/fcimb.2019.00049 (PMC6422106; doi:10.3389/fcimb.2019.00049)
Supplement: Supplementary file 1 [file Data_Sheet_1.PDF]

frontiers

Supplementary Material

Activity and impact on resistance development of two antivirulence fluoropyrimidine drugs in *Pseudomonas aeruginosa*

Francesco Imperi[1,2*], Ersilia Fiscarelli[3], Daniela Visaggio[1], Livia Leoni[1], and Paolo Visca[1]

[1] Department of Science, Roma Tre University, Rome, Italy
[2] Department of Biology and Biotechnology Charles Darwin, Sapienza University of Rome, Laboratory affiliated to Istituto Pasteur Italia – Fondazione Cenci Bolognetti, Rome, Italy
[3] Laboratory of Cystic Fibrosis Microbiology, Bambino Gesú Hospital, Rome, Italy

*Correspondence: Francesco Imperi, Department of Science, University "Roma Tre", Viale Marconi 446, Rome, 00146, Italy. francesco.imperi@uniroma3.it

1. Supplementary Figures and Tables

1.1. Supplementary Figures

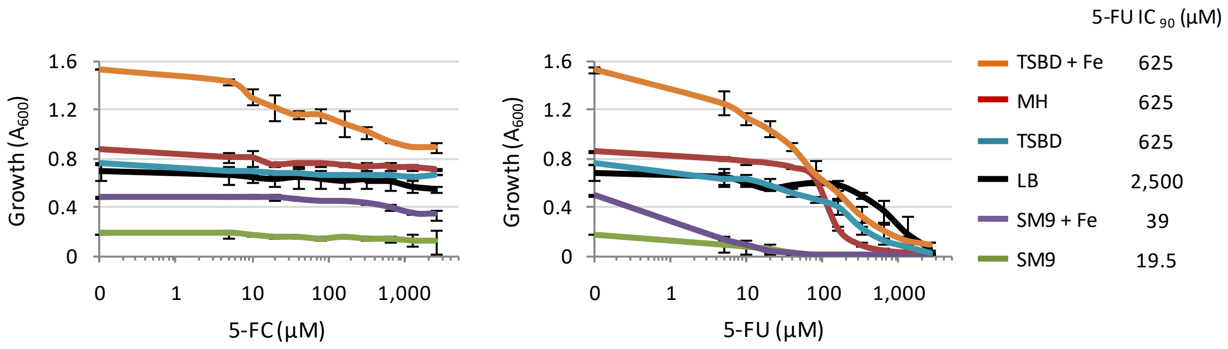

Supplementary Figure 1. Effect of (A) 5-fluorocytosine (5-FC) and (B) 5-fluorouracil (5-FU) (0-2,500 µM) on *P. aeruginosa* PAO1 growth in different media, supplemented or not with 100 µM $FeCl_3$ (+ Fe) after 18-h growth in microtiter plates at 37°C. Abbreviations: MH, Mueller-Hinton broth; TSBD, trypticase soy broth dialysate; LB, Luria-Bertani broth; SM9, M9 minimal medium supplemented with succinate. Growth (OD_{600}) was measured in a microtiter plate reader, and values represent the mean (± SD) of three independent assays.

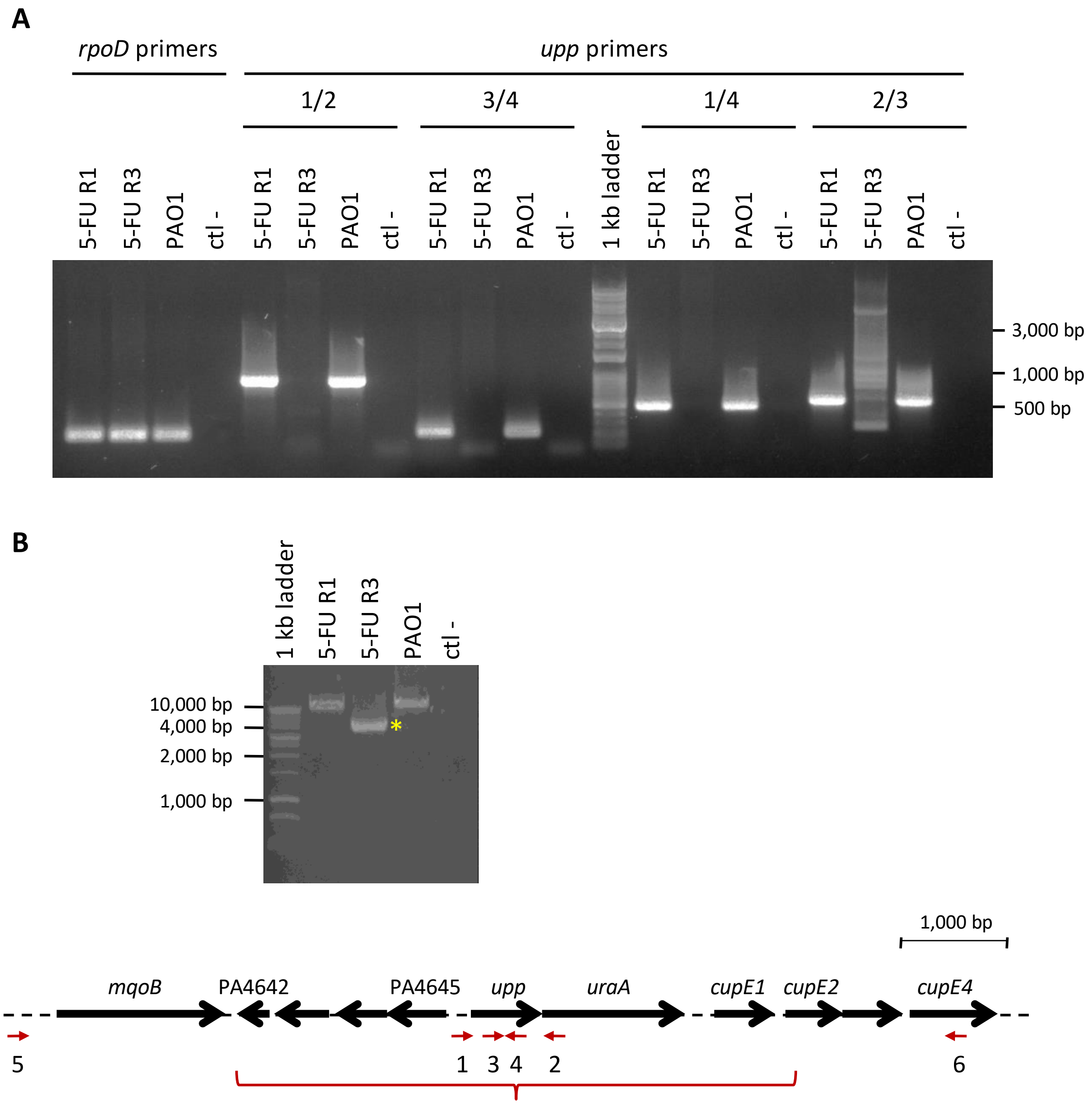

Supplementary Figure 2. (A) PCR analysis showing the lack of the expected amplicons for the spontaneous mutant R3 with four different primer pairs annealing inside and/or just outside the *upp* gene. The expected amplicons were instead obtained for the spontaneous mutant R1 and the wild type PAO1, used as positive controls. A PCR with primers annealing inside the essential *rpoD* gene (Supplementary Table 1) were used to confirm the integrity of the R3 genomic DNA. **(B)** Long-range PCR with LA Taq DNA polymerase (TaKaRa) using primer pairs 5/6 and the genomic DNA of strains R1, R3 or PAO1 as the template. The PCR product of ca. 4 kb (highlighted with a yellow asterisk) was purified from gel and partially sequenced. Sequencing of the amplicon revealed a 5.1-kb deletion including the *upp* gene, the neighbouring genes PA4642-PA4645, *uraA*, *cupE1* and ca. 25% of *cupE2*. A scheme reporting the annealing position of the primers used in these assays is shown below the figure, where the red curly bracket indicates the deletion in the R3 mutant. Primer abbreviations: 1, upp FW_seq; 2, upp RV_seq; 3, upp_RT_FW; 4, upp_RT_RV; 5, upp locus_FW; 6, upp locus_RV (Supplementary Table 1).

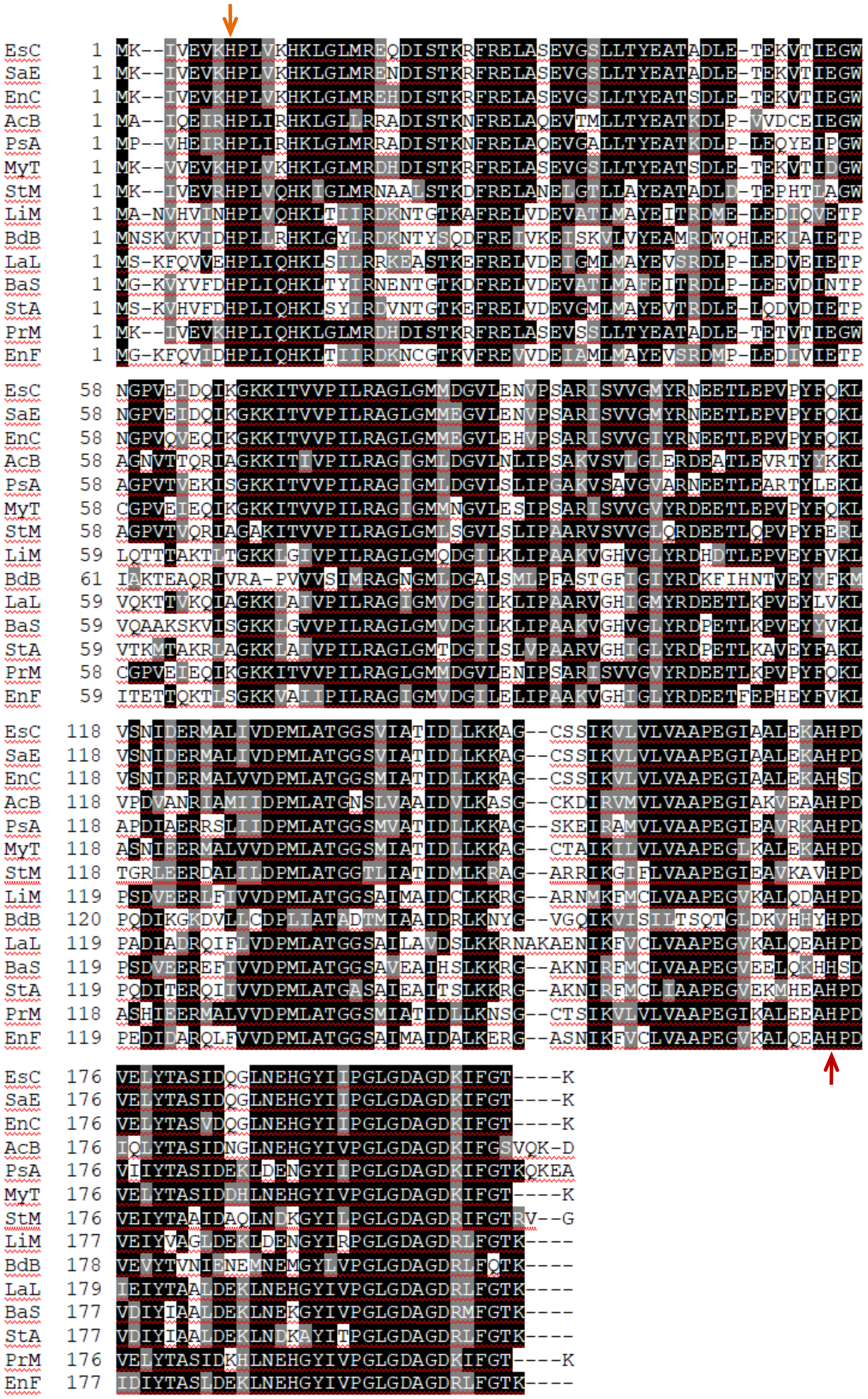

Supplementary Figure 3. Alignment of Upp proteins from different eubacterial species. Abbreviations: EsC, *Escherichia coli*; SaE, *Salmonella enterica* serovar Typhimurium; EnC, *Enterobacter cloacae*; AcB, *Acinetobacter baumannii*; PsA, *Pseudomonas aeruginosa*; MyT, *Mycobacterium tuberculosis*; StM, *Stenotrophomonas maltophilia*; LiM, *Listeria monocytogenes*; BdB, *Bdellovibrio bacteriovorus*; LaL, *Lactococcus lactis*; BaS, *Bacillus subtilis*; StA, *Staphylococcus aureus*; PrM, *Proteus mirabilis*; EnF, *Enterococcus faecium*. The red and orange arrows highlight the histidine residues which are mutated into proline in the spontaneous 5-FU resistant mutant R1 and in R2/R4, respectively (Supplementary Table 1).

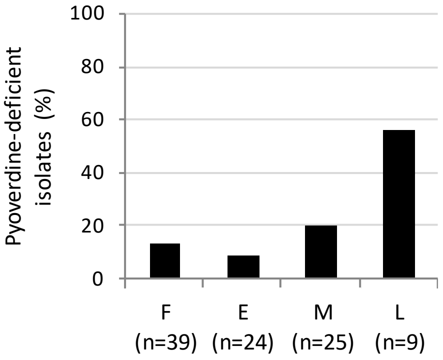

Supplementary Figure 4. Percentage of pyoverdine-deficient strains in *P. aeruginosa* CF isolates grouped on the basis of the duration of the chronic lung infection (F, first isolates; E, early-stage isolates; M, middle-stage isolates; L, late-stage isolates). The number of isolates included in each group is reported below the graph.

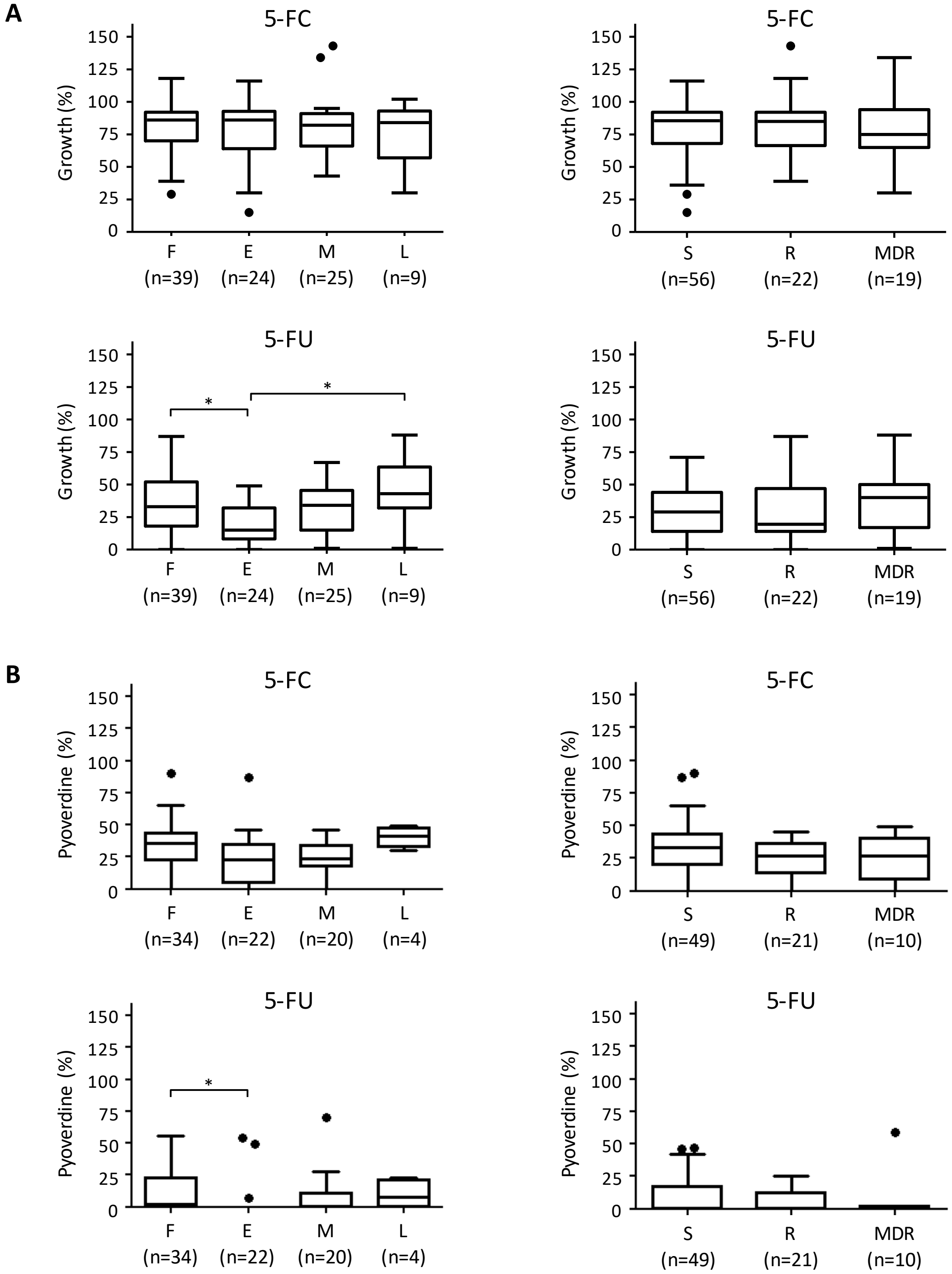

Supplementary Figure 5. Box plots with Tukey whiskers showing the effect of 5-fluorocytosine (5-FC) and 5-fluorouracil (5-FU) at 100 μM on growth (panel A) and pyoverdine production (panel B) of *P. aeruginosa* CF isolates, shown as percentage relative to untreated cultures (100%). Isolates were clustered on the basis of the duration of the chronic lung infection (left panels) or of the antibiotic resistance profile (right panels). Black dots represent the outliers. Asterisks indicate statistically significant differences (P< 0.05; Kruskal-Wallis). Abbreviations: F, first isolates; E, early-stage isolates; M, middle-stage isolates; L, late-stage isolates; S, sensitive; R, resistant to 1-2 antibiotics belonging to different classes; MDR, multi-drug resistant, *i.e.* resistant to ≥ 3 antibiotics belonging to different classes (see Supplementary Table 2 for details). The number of isolates included in each group is reported below the graphs.

1.2. Supplementary Table

Supplementary Table 1. Bacterial strains, plasmids and primers used in this study.

**Strain**	**Genotype and/or relevant characteristics**	**Reference/source**
P. aeruginosa		
PAO1 (ATCC15692)	Wild type (prototroph)	American Type Culture Collection
PAO1 5-FU R1	Spontaneous 5-FU resistant mutant of PAO1, carrying a missense point mutation (518A>C) in the *upp* gene leading to the H173P amino acid substitution	This work
PAO1 5-FU R2	Spontaneous 5-FU resistant mutant of PAO1, carrying a missense point mutation (23A>C) in the *upp* gene leading to the H8P amino acid substitution	This work
PAO1 5-FU R3	Spontaneous 5-FU resistant mutant of PAO1, carrying a deletion involving the *upp* gene	This work
PAO1 5-FU R4	Spontaneous 5-FU resistant mutant of PAO1, carrying a missense point mutation (23A>C) in the *upp* gene leading to the H8P amino acid substitution	This work
PAO1 5-FU R5	Spontaneous 5-FU resistant mutant of PAO1, carrying a nonsense point mutation (170G>A) in the *upp* gene leading to the W57X codon mutation	This work
PAO1 Δ*pvdA*	PAO1 derivative with a deletion of the *pvdA* gene	Imperi *et al.*, 2008
E. coli		
DH5αF'	*recA1 endA1 hsdR17 supE44 thi-1 gyrA96 relA1* Δ(*lacZYA-argF*)U169 [Φ*80*d*lacZ*ΔM15], Nal^R	Liss, 1987

**Plasmid**	**Relevant characteristics**	**Reference**
pUCP18	*E. coli-Pseudomonas* shuttle vector, Ap/Cb^R	Schweizer, 1991
pUCP*codA*	pUCP18 derivative carrying the entire *codA* coding sequence under the control of the P_{T5} *lacO* region	Imperi *et al.*, 2013
pUCP*codB*	pUCP18 derivative carrying the entire *codB* coding sequence under the control of the P_{T5} *lacO* region	Imperi *et al.*, 2013
pUCP*codAcodB*	pUCP18 derivative carrying the entire *codA* and *codB* coding sequences under the control of the P_{T5} *lacO* region	This work
pUCP*upp*	pUCP18 derivative carrying the *upp* gene, including its own promoter	This work

**Primer**	**Sequence (restriction site[a])**	**Application**
upp_pUCP18_FW	5'-cggaaTTCTCCCTGGCTGGGCTG-3' (EcoRI)	Cloning of *upp* in
upp_pUCP18_RV	5'-cgcaagctTCCTGGAAGGCATCGCCC-3' (HindIII)	pUCP18
upp FW_seq	5'-GACATGGTGCTTCTCCCTG-3'	Amplification and
upp RV_seq	5'-AGGCGACGAACAGCATCT-3'	sequencing of *upp*
upp_RT_FW	5'-CTGCTGACCTACGAAGCGA-3'	Amplification of *upp*
upp_RT_RV	5'-CGTGCTTCCAGGGTCTCTT-3'	(internal region)
upp locus_FW	5'-TGTGGCGAGCAATGAAGAGT-3'	Amplification and
upp locus_RV	5'-ACAGCGGTACCGAATAGCG-3'	sequencing of the *upp* genomic locus
upp locus_FW2	5'-TGGACGTACATGGGAACCTC-3'	Sequencing of the *upp* genomic locus
rpoD_RT_FW	5'-GGGCGAAGAAGGAAATGGTC-3'	Amplification of *rpoD*
rpoD_RT_RV	5'-CAGGTGGCGTAGGTGGAGAA-3'	(internal region)

[a] The restriction site is underlined in the primer sequence.

Additional references (not included in the main text)

Imperi, F., Putignani, L., Tiburzi, F., Ambrosi, C., Cipollone, R., Ascenzi, P., *et al.* (2008). Membrane-association determinants of the omega-amino acid monooxygenase PvdA, a pyoverdine biosynthetic enzyme from *Pseudomonas aeruginosa*. Microbiology 154, 2804-2813. doi: 10.1099/mic.0.2008/018804-0.

Liss, L. (1987). New M13 host: DH5 F′ competent cells. Focus 9, 13.

Schweizer, H.P. (1991). *Escherichia-Pseudomonas* shuttle vectors derived from pUC18/19. Gene. 97, 109-121.

Supplementary Table 2. General features, antibiotic resistance profile and 5-FU/5-FC sensitivity of the 100 CF strains analysed in this study.

Isolate[1]	Years of CF lung colonization	Antibiotic resistance profile[2]										Growth (% relative to NT cultures)[3]				Pyoverdine (% relative to NT cultures)[3]			
		PIP/TAZ	ATM	CAZ	MEM	IMP	AK	TM	CIP	LV	COL	5-FC		5-FU		5-FC		5-FU	
												10 µM	100 µM	10 µM	100 µM	10 µM	100 µM	10 µM	100 µM
BG 1	first isolate	S	I	S	S	S	S	R	S	I	S	96	97	85	42	34	21	19	3
BG 2	3	S	I	S	S	S	R	R	S	S	S	96	91	102	49				
BG 3	6	R	R	R	S	S	S	R	S	S	S	80	71	83	50	14	11	10	2
BG 4	first isolate	S	I	S	S	S	S	S	S	S	S	86	78	66	22	26	33	22	0
BG 5	2	S	I	S	S	S	S	R	S	S	S	74	71	66	5	20	12	9	0
BG 6	6	S	S	S	S	S	S	R	S	S	S	90	86	70	21	20	17	8	0
BG 7	first isolate	S	I	S	S	S	S	S	S	S	S	97	88	89	54	57	51	46	19
BG 8	2	S	I	S	S	S	R	S	S	S	S	71	64	59	8	33	36	33	0
BG 9	5	S	S	S	S	S	S	S	S	S	S	67	64	52	6	34	30	27	0
BG 10	first isolate	S	I	S	S	S	S	S	S	S	S	88	81	61	29	36	22	31	0
BG 11	3	S	R	S	S	S	S	S	S	S	S	95	87	62	46	44	32	48	6
BG 12	5	S	S	S	S	S	S	S	S	S	S	86	84	54	41	29	20	9	0
BG 13	first isolate	R	R	R	R	R	S	R	I	I	S	89	83	69	19	36	36	28	0
BG 14	3	S	I	R	R	R	S	R	S	I	S	74	67	74	7	51	40	10	0
BG 15	5	S	I	R	R	R	R	R	S	S	S	147	134	126	17	37	32	21	2
BG 16	first isolate	S	I	S	S	S	S	S	S	S	S	99	96	94	71	52	38	51	34
BG 17	2	S	I	S	S	S	R	S	S	S	S	129	116	102	29	14	8	6	0
BG 18	6	S	S	S	S	S	R	S	S	S	S	103	92	99	43	21	17	18	13
BG 19	first isolate	S	S	S	I	R	R	R	S	S	S	92	70	88	16	23	22	19	1
BG 20	3	S	S	S	R	R	R	R	S	S	S	66	64	51	8	8	8	2	0
BG 21	5	S	S	S	R	R	R	R	S	S	S	92	89	86	36	26	21	37	0
BG 22	first isolate	S	S	S	S	S	S	S	S	S	S	104	99	90	18	60	58	41	0
BG 23	2	S	S	S	S	S	S	S	S	S	S	99	94	82	14	42	35	23	0
BG 24	first isolate	S	S	R	R	R	R	R	R	R	S	82	75	56	7	18	15	12	0
BG 25	2	S	S	R	R	R	R	R	R	R	S	34	30	17	17	0	0	0	0
BG 26	5	R	R	R	R	R	R	R	R	R	S	66	63	35	14	13	22	28	59
BG 27	first isolate	S	I	S	S	S	S	S	S	S	S	92	88	78	29	32	24	22	2
BG 28	2	S	I	S	S	S	S	S	S	S	S	88	86	75	21	39	31	32	0
BG 29	5	S	S	S	S	S	S	S	R	R	S	80	67	56	14	33	31	24	0
BG 30	first isolate	S	I	S	S	S	S	S	S	S	S	85	78	79	54	86	90	72	47
BG 31	3	S	I	S	S	S	S	S	S	S	S	78	64	69	15	52	46	38	0
BG 32	5	S	I	S	S	S	S	S	S	S	S	79	68	74	19	55	46	39	0
BG 33	first isolate	S	I	S	S	S	R	S	S	S	S	55	39	16	0	0	0	0	0
BG 34	2	S	I	S	S	S	R	R	S	S	S	91	86	80	15	34	27	24	0
BG 35	first isolate	S	I	S	S	S	S	S	S	S	S	80	71	50	6	21	17	9	0
BG 36	3	S	S	S	S	S	S	S	S	S	S	103	93	58	9	11	5	0	0
BG 37	first isolate	S	I	S	S	S	S	R	S	S	S	103	95	84	17	29	29	17	0
BG 38	3	S	S	S	S	S	R	R	I	S	S	97	92	81	14	29	18	13	0
BG 39	first isolate	S	I	S	S	S	S	S	S	S	S	95	92	90	20				
BG 40	3	S	S	S	I	R	S	S	S	S	S	90	86	61	18	0	0	0	0
BG 41	first isolate	S	I	S	S	S	S	S	S	S	S								
BG 42	3	S	I	S	R	R	S	S	S	S	S	91	83	67	4	45	32	28	0
BG 43	first isolate	S	I	S	S	S	S	S	S	S	S	90	83	63	3	74	58	22	0
BG 44	3	S	S	R	S	S	R	R	S	S	S	81	110	51	33				
BG 45	first isolate	S	I	S	S	S	S	S	S	S	S	36	29	38	3	0	0	0	0
BG 46	2	S	I	S	S	S	S	S	S	S	S	29	15	32	0	0	0	0	0
BG 47	first isolate	R	I	S	S	S	S	R	S	S	S	71	65	53	21	3	0	0	0
BG 48	first isolate	S	I	S	S	S	S	S	S	S	S	87	82	76	35	41	34	29	9
BG 49	2	S	I	S	S	S	S	S	S	S	S	35	36	41	20	40	25	29	42
BG 50	first isolate	S	I	S	S	S	S	S	S	S	S	77	73	78	7	46	41	47	0
BG 51	2	S	I	S	S	S	S	S	R	I	S								
BG 52	first isolate	S	I	S	S	S	S	S	S	S	S	87	66	71	50	51	30	35	19
BG 53	2	S	I	S	S	S	S	S	S	S	S	93	76	71	14	29	21	12	0
BG 54	first isolate	S	I	S	S	S	S	S	S	S	S	112	92	66	44	68	65	47	18
BG 55	3	S	S	S	S	S	S	S	S	S	S	100	96	47	38	88	87	58	46
BG 56	first isolate	S	I	S	S	S	R	R	I	I	S	96	64	96	44	38	37	17	1
BG 57	first isolate	S	I	S	I	R	S	S	S	S	S	112	118	116	87	48	37	32	25
BG 58	2	S	I	S	S	S	S	S	S	S	S	89	87	78	34	6	2	2	0
BG 59	first isolate	S	I	S	S	S	S	S	S	S	S	90	86	84	71	53	39	39	30
BG 60	first isolate	S	S	S	S	S	S	S	S	S	S	116	101	111	52	38	36	31	19
BG 61	2	S	I	S	S	S	S	S	S	S	S	76	62	73	13	10	0	10	0
BG 62	first isolate	S	I	S	S	S	S	S	S	S	S	100	89	80	66	40	36	37	24
BG 63	2	S	I	S	S	S	S	S	S	S	S	89	95	68	44	22	37	11	0
BG 64	first isolate	S	I	S	S	S	S	S	S	S	S	64	56	52	11				
BG 65	first isolate	S	I	S	S	S	S	S	S	S	S	72	68	60	33				
BG 66	first isolate	S	S	S	S	S	S	S	S	S	S	105	103	86	56	41	28	29	17
BG 67	first isolate	S	S	S	S	S	S	S	S	S	S	102	87	78	34				
BG 68	first isolate	S	I	S	S	S	S	S	S	S	S	93	91	75	25	48	51	29	0
BG 69	first isolate	S	I	S	S	S	S	S	S	S	S	95	91	80	60	52	44	22	6
BG 70	first isolate	S	I	S	S	S	S	S	S	S	S	94	95	84	33				
BG 71	first isolate	S	I	S	S	S	S	S	S	S	S	95	91	78	64	50	43	24	11
BG 72	first isolate	S	I	S	S	S	S	S	S	S	S	75	84	80	25	63	44	37	0
BG 73	first isolate	S	I	S	S	S	S	S	S	S	S	101	95	97	58	62	55	43	36
BG 74	first isolate	S	I	S	S	S	S	S	S	S	S	67	59	62	41	48	42	36	26
BG 75	first isolate	S	I	S	S	S	S	S	S	S	S	68	58	57	32	28	19	10	2
BG 76	7	S	I	R	S	R	I	S	S	S	S	53	45	26	16	4	2	0	0
BG 77	6	R	R	R	R	R	R	R	I	I	S	96	92	83	67				

BG 78	5	S	S	R	R	R	S	R	R	R	S	84	76	73	40					
BG 79	7	S	I	S	S	S	S	R	S	S	S	100	90	116	58	24	21	9	0	
BG 80	5	S	S	S	S	S	R	S	I	R	S	85	80	75	63	52	45	37	24	
BG 81	5	S	I	S	S	S	S	S	I	I	S	60	47	55	7					
BG 82	6	S	I	S	R	S	I	R	R	R	S	69	65	59	38	5	0	4	0	
BG 83	5	I	R	S	S	S	S	R	S	S	S	90	82	86	56	39	36	42	22	
BG 84	5	I	I	S	S	S	R	S	S	S	S	138	143	128	34	32	25	17	12	
BG 85	6	S	S	S	R	S	R	R	S	S	S	92	86	28	2	41	38	0	0	
BG 86	6	S	S	S	S	S	S	S	S	S	S	83	78	51	31	52	33	25	1	
BG 87	6	S	S	S	S	S	S	S	S	S	S	56	43	14	1	31	0	0	0	
BG 88	5	S	S	R	R	R	R	I	S	S	S	91	95	112	43					
BG 89	6	S	S	S	R	R	R	I	R	R	S	86	95	77	48					
BG 90	7	S	S	S	S	S	S	S	S	S	S	90	85	74	23	43	35	28	0	
BG 91	≥ 15	R	R	R	R	R	R	R	R	R	S	65	48	48	21					
BG 92	≥ 15	R	R	R	R	R	R	R	R	R	S	77	102	80	68	90	49	128	0	
BG 93	≥ 15	S	S	S	R	R	S	R	R	R	S	76	66	85	57					
BG 94	≥ 15	R	R	R	R	R	R	R	R	R	S									
BG 95	≥ 15	S	I	R	R	R	R	I	R	R	S	107	94	87	88					
BG 96	≥ 15	S	S	S	S	S	R	R	S	S	S	99	84	87	59	37	30	25	13	
BG 97	≥ 15	R	R	R	R	R	R	R	R	R	S	33	30	37	1					
BG 98	≥ 15	S	S	S	S	S	R	R	R	R	R	75	68	73	43	77	43	78	0	
BG 99	≥ 15	S	R	R	R	R	R	I	S	S	S	90	92	88	43					
BG 100	≥ 15	S	I	S	S	S	R	R	R	R	S	90	92	88	43	62	39	40	20	
												85	79	72	31	36	29	25	8	Mean
												20	21	22	21	21	19	21	13	SD

[1] Strains with the same background colour correspond to sequential isolates from the same patient. When indicated strains were grouped in four categories on the basis of the years of lung colonization [F, first isolates; E, early-stage isolates (2-3 years since the first isolation); M, middle-stage isolates (5-7 years since the first isolation); L, late-stage isolates (>15 years since the first isolation)].

[2] S, sensitivity; I, intermediate resistance; R, resistance (according to EUCAST criteria). Antibiotic abbreviations: PIP/TAZ, piperacillin/tazobactam; ATM, aztreonam; CAZ, ceftazidime; MEM, meropenem; IMP, imipenem; AK, amikacin; TM, tobramycin; CIP, ciprofloxacin; LVX, levofloxacin; COL, colistin.

[3] Empty and light grey cells indicate strains that do not grow and/or that do not produce detectable levels of pyoverdine in TSBD medium. Red and green cells highlight strains with values lower that the mean - SD and higher than the mean + SD calculated for each specific condition, respectively (corresponding to strains more sensitive and more resistant than the average of the strain collection, respectively).
